# The Role of Citrullinated Protein Antibodies in Predicting Erosive Disease in Rheumatoid Arthritis: A Systematic Literature Review and Meta-Analysis

**DOI:** 10.1155/2015/728610

**Published:** 2015-03-04

**Authors:** A. A. Jilani, C. G. Mackworth-Young

**Affiliations:** ^1^Chelsea and Westminster NHS Foundation Trust, 369 Fulham Road, London SW10 7NH, UK; ^2^Charing Cross Hospital, Imperial College Healthcare NHS Trust, Fulham Palace Road, London W6 8RF, UK

## Abstract

*Background*. Autoantibodies to citrullinated peptides have been shown to be valuable in the diagnosis of rheumatoid arthritis (RA). The expanding repertoire of antibodies to citrullinated peptide antigens (ACPA) has been a topic of great interest in recent reviews and research studies, as has the ability of these autoantibodies to predict disease outcome. *Objectives*. The aim of this review was to provide an update on the relevance of ACPA as prognostic markers in RA. The ability to identify patients predisposed to an aggressive outcome at the time of initial diagnosis greatly facilitates the selection of appropriate and cost-effective treatment. *Methods*. A systematic review of the literature was carried out. Studies from 1967 up to June 2014 with data on prognostic value of ACPA were included. Quality assessment was done by using the modified Hayden list for prognostic studies. Meta-analysis was performed using BioStat software. *Results*. The results of 25 studies were selected for the final review. A total of 6421 patients with RA were included, mainly in inception cohorts, with follow-up duration ranging from one year to ten years. All studies carried prognostic data on all available isotypes of anticyclic citrullinated protein (CCP), while four had data on antimutated citrullinated vimentin (MCV). There was a single relevant study each on anticitrullinated enolase peptide 1 (CEP1) and antichimaeric fibrin/filaggrin citrullinated peptide 1 (CFFCP1). All studies showed ACPA to be strong predictors of joint erosions in RA. Other factors, particularly baseline erosions, showed an additive effect. Anti-MCV appeared to be a marker of a more aggressive form of disease. Ten studies had data on which a meta-analysis could be performed. This gave an overall odds ratio of 4.85 for ACPA (anti-CCP/MCV) positivity being predictive for the development of joint erosions. Two studies with data on anti-CEP1 and anti-CFFCP1 also showed this positive predictive role of ACPA for joint erosions. *Conclusions*. ACPA are strong predictors of severity in RA. Their use should be part of routine rheumatology practice.

## 1. Introduction

Rheumatoid arthritis (RA) is a heterogeneous condition. This is well illustrated by the highly variable course the disease may follow in different individuals. An important potential outcome is the development of joint damage, in particular articular erosions. These develop rapidly in a quarter of patients with RA within three months of onset, and about three-quarters develop erosions within the first two years of being diagnosed [1]. In the early stages of disease it is difficult to predict which patients will develop rapidly progressive joint damage. This is clinically important, since it is now well established that early intervention in RA improves the overall prognosis of the disease. Disease-modifying drugs have potentially severe side effects. Clinicians therefore need the tools to initiate therapy early in patients likely to have an adverse outcome.

Some indicators of poor prognosis have been known for many years: for instance, a strongly positive rheumatoid factor (RF) test may be predictive of severe disease, as may the presence of the so-called “shared epitope” (HLA DRB^**^01, 04) [2, 3]. We know that there is also a role played by genetics, in particular PTNP22 genotype, and environmental factors such as smoking, infection with* Porphyromonas gingivalis*, the use of the oral contraceptive pill, and high caffeine intake. They are all risk factors for developing RA. However the predictive value of these environmental factors is relatively weak.

The discovery of antibodies to citrullinated protein antigens (ACPA) represented a turning point in the management of this condition. Initially the presence of these antibodies was discovered to be particularly useful diagnostically: it was more specific, although less sensitive, than the presence of rheumatoid factor. Early studies also suggested that individuals with ACPA were more likely to develop severe disease [4, 5].

The presence of citrullinated proteins in the joint synovium is not specific for RA. Other causes of inflammation and systemic infections can result in citrullination of synovial peptides [5, 6]. The development of ACPA however has been shown to have a high specificity for RA [3–5].

The citrullination of arginine to citrulline as a result of deamination by peptidyl arginine deiminase (PAD) is a physiological process that takes place during cell apoptosis. More specifically polymorphisms within PAD 2 and PAD 4 appear to impart an increased susceptibility to RA [7, 8]. The synovium in RA contains many citrullinated proteins. These include citrullinated fibrin, citrullinated vimentin, citrullinated alpha enolase, and citrullinated collagen type II. Antibodies to all of these have specificity for RA.

Bang et al. discovered an isoform of vimentin in which glycine replaces arginine residues and named it mutated or modified vimentin [9]. Recent work has shown antibodies to this specificity of ACPA to have high diagnostic accuracy and a potent predictive capability [10].

This review was carried out to examine the current literature on the value of ACPA positivity in predicting erosive damage to joints and, second, to evaluate the potential role of different ACPA specificities in predicting this erosive damage, focussing on comparing anti-CCP positivity with anti-MCV positivity.

## 2. Methods

This review followed the Cochrane principles for systematic reviews and recommendations for assessing prognostic studies. All study types were included which assessed the value of ACPA to predict joint damage in RA.

### 2.1. Inclusion Criteria

A literature search was carried out using the Cochrane Library, Embase, the Centre for Evidence-Based Medicine at Oxford, and Medline, via PubMed and Bandolier, for abstracts and papers from 1967 up to June 2011. A free text search was carried out using “anti-CCP,” “citrullinated proteins,” “rheumatoid arthritis,” and “joint erosion” as search terms together with all synonyms. All observational studies which compared patients with RA and controls were considered for inclusion. These included cohort studies and case-control studies. The studies all included adults aged 18 or more with a clinical diagnosis of RA [11, 12]. Studies could include patients with undifferentiated inflammatory arthritis (UIA), but those involving juvenile arthritis and other rheumatic diseases were excluded. Studies could include healthy controls.

Studies were included that presented primary data that directly addressed the prognostic relevance of ACPA in RA, specifically the role of these antibodies in prediction of aggressive disease leading to joint erosions. Studies had to describe or refer to the methods used to analyse ACPA and to include measurement of radiological joint damage by either the Sharp scoring system, as modified by van der Heijde (SvdH) [13], the Larsen score [14], or the simple erosion narrowing (SEN) score [15].

### 2.2. Methodological Quality of Included Studies

The quality of the studies was assessed using the modified Hayden list for prognostic studies [16]. This included the accurate description of the study population, the stage of disease, the rationale for the size of cohort, and blinding at the time of X-ray interpretation. Studies were assessed according to the Hayden list to highlight sources of bias. The studies were graded from zero to six according to their adherence to this [16]. Studies that adhered to at least four of these six requirements to assess study bias were included.

### 2.3. Data Extraction and Analysis

Data extracted from each trial included the duration of study, number of patients recruited, the number that completed the trial, the types of ACPA studied, and the kits used for analysis.

Ten studies had data computable for a meta-analysis. These were pooled to derive the outcome measures used for a forest plot of the data. The odds ratio of developing joint erosions in ACPA positive patients was calculated using BioStat software. The likelihood ratio of developing joint erosions was calculated using the SvdH, Larsen, or SEN scores.

## 3. Results

### 3.1. Studies Included

**Figure 1:**
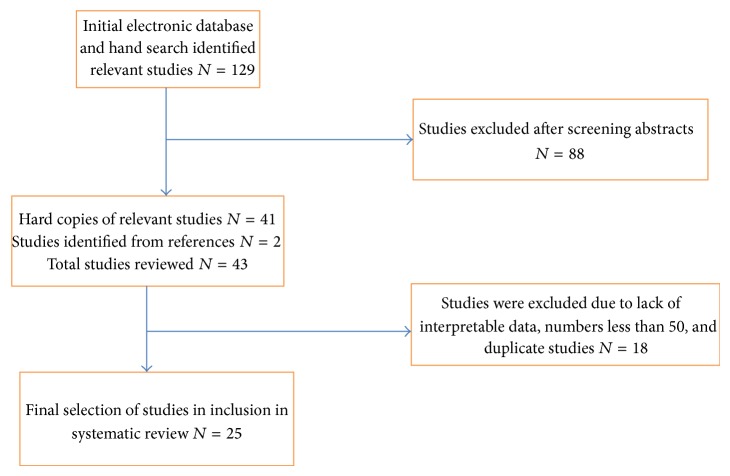
Study Selection for systematic review.

The initial electronic search and hand search identified 129 relevant studies. 88 were excluded after a review of the abstract. Hard copies of 41 studies were obtained for further review; two further studies were included from the reference lists of these papers that had not been identified by the electronic and hand searches. After quality screening for relevance and interpretable data and the study design used 25 studies were selected for this systematic review (Figure 1). These 25 studies (Table 1) had enrolled 6,421 patients (range 55–872). Their duration ranged from one to ten years. Twenty-two studies used the revised 1987 ACR criteria for the diagnosis of RA [11]; one used the 1958 ARA criteria [12]; and two fulfilled both sets of criteria.

### 3.2. Radiographic Assessment

All included studies looked at X-rays of hands and feet. Four studies also looked at wrists, one at elbows, and one at axial involvement (particularly cervical spine). All studies used two investigators for reading the X-rays independently and blind to other patient data. In 23 studies X-ray films were read by radiologists; in two studies they were read by rheumatologists trained in reading radiographs. In all but one study erosions were scored using the SvdH or Larsen score. The remaining study used the SEN score. Although all studies used conventional radiographs, two studies also used MRI scanning to detect joint damage. The MRI data was not used for analysis.

### 3.3. ACPA Analysis

Most studies used the anti-CCP2 test for ACPA analysis. The majority (*n* = 17) used the Eurodiagnostica kit to analyse this (normal range 0–25 U/mL; sensitivity 76.5%). Four studies used the Inova Diagnostics kit (normal range 0–25 U/mL; sensitivity 82%), and another four used the Axis-Shield kit (normal range 0–5 U/mL sensitivity 88%). Anti-MCV was analysed in six papers. All of these used the ELISA by Orgentec Diagnostics with a positive cutoff at 20 U/L. One study used the western blot technique and an in-house ELISA using bovine MBP (myelin basic protein). The overall sensitivity for anti-CCP was 82% with a specificity of 96%. For anti-MCV the sensitivity was 80% with a specificity of 97%. The sensitivity and specificity for anti-CFFCP 1 were 83% and 97%, respectively.

### 3.4. Meta-Analysis

**Figure 2:**
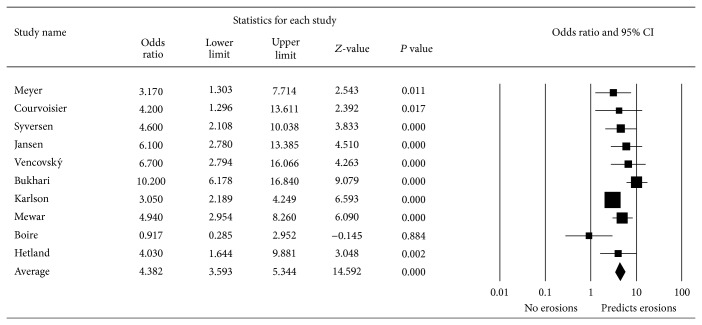
Meta-analysis of ACPA as predictors of erosions in rheumatoid arthritis.

Ten studies had data on which a meta-analysis was computable. Of the studies that used anti-MCV, only three had data on which a meta-analysis was computable. Due to the small number these were not considered for a separate analysis (Figure 2). The selected studies included seven prospective trials, a longitudinal study, a cross-sectional trial, and a double-blind randomized control trial. A total of 3065 patients were enrolled. The mean study duration was 4.7 years. For nine studies there was an odds ratio greater than one for ACPA predicting the development of erosions. There was an overall odds ratio for all ten studies of 4.38 within 95% CI (5.34–3.59). One study that enrolled 165 patients over two years failed to show this positive trend of ACPA to predict joint erosions. Instead it found the presence of the shared epitope and baseline erosions to be more significant predictors of joint damage.

### 3.5. Individual Prognostic Indicators

#### 3.5.1. Anti-CCP Antibodies

Two studies recruiting 55 and 254 patients followed up over three and five years, respectively, show by regression analysis the presence of anti-CCP at baseline to be highly predictive of erosive disease [17, 18]. Bukhari et al. recruited 254 patients from the Norfolk Arthritis Register and found the presence of ACPA at baseline was strongly associated with developing erosions over a period of 5 years [18]. This association was more significant in RF-negative patients. The ability of ACPA to predict erosions was also illustrated in the study by Kaltenhäuser et al. They evaluated the predictive value of ACPA for joint erosion in 126 patients prospectively over six years [19]. ACPA positive patients had significantly higher Larsen scores at all-time points analysed in this study. A mean Larsen score of 28 was reported after six years in the anti-CCP positive as compared to 19 in the anti-CCP negative patients.

In a cohort of 157 patients with RA Rojas-Villarraga et al. used the SvdH for scoring erosions over a period of three years [20]. They showed the hazard of appearance of substantial joint damage was 99% higher in patients who were anti-CCP positive than those without the autoantibody.

#### 3.5.2. Anti-MCV Antibodies

Five cohort studies, four of these with controls, analysed anti-MCV and found these autoantibodies to be predictive of a more aggressive form of disease as measured by SvdH or Larsen scores.

Two studies directly compared anti-MCV and anti-CCP antibodies. Syversen et al. showed that anti-MCV increased the odds of radiographic progression by 7.3 (95% CI 3.2 to 16.5) compared to 5.7 (95% CI 2.6 to 12.5) for positive anti-CCP [21]. The overall increase in SvdH score was 30 for the anti-MCV positive and 25 for the anti-CCP positive patients. Mathsson et al. recruited 273 patients with early RA and found anti-MCV to have a higher predictive value as compared to anti-CCP for the development of joint erosions [22].

van der Linden et al. [29] analysed the predictive value for joint erosion of RF, anti-CCP-2, anti-CCP-3, and anti-MCV in 687 patients in a five-year longitudinal study. They compared single tests and combinations of these tests. All four tests individually showed comparable associations with the rate of joint destruction. There was no statistical difference among the four tests with regard to their ability to predict erosions. The presence of either two or three of these autoantibodies was associated with a higher rate of joint erosions as compared to a single antibody. In patients with ACPA the additional presence of RF was not significantly associated with an enhanced rate of joint destruction.

Bukhari et al. studied a cohort of 165 patients prospectively, looking at RF, anti-CCP, and anti-Sa (anti-MCV) [18]. Of these they found that anti-Sa were the best predictors of disease severity. Further multivariate analysis showed the presence of anti-Sa (OR 8.83), baseline erosions (OR 3.47), and increasing age (OR 1.06/year) to be significantly associated with disease severity. Finally Mansour et al. described changes in the axial skeleton detected on MRI scanning and also peripheral joint damage on X-rays in a prospective cohort of 64 RA patients and 59 controls with other rheumatic diseases over two years [24]. Anti-MCV was a strong predictor of joint damage: patients had significantly higher SEN scores when anti-MCV positive. The study did not look at other types of ACPA.

#### 3.5.3. Other ACPA Specificities

Two studies analysed ACPA other than anti-CCP and anti-MCV. One of these studied the role of anti-CFFCP1 as a prognostic marker [28]. Three subtypes of anti-CFFCP were analysed. Of these anti-CFFCP1 best identified patients with a poor radiographic outcome: the authors reported greater radiographic progression in anti-CFFCP1-positive patients independent of their anti-CCP status. The mean Larsen score progressed from 1.3 at entry to 6.0 at the end of follow-up. The other study examined anti-CEP1 in comparison to anti-CCP in 408 patients from the NOAR cohort over a five-year follow-up and found no statistical difference between anti-CCP2 and anti-CEP1 in predicting radiological damage [23]. No studies directly investigated whether higher ACPA levels of any specificity are associated with greater radiological progression than lower levels.

#### 3.5.4. Other Prognostic Indicators

Five studies [17, 19, 30–32] showed a positive predictive value of the shared epitope for the development of erosive damage, with the presence of ACPA also a strong predictor except in one study. In this inception cohort of 134 patients with recent onset RA, studied over a year, Reneses et al. found homozygous SE status and the presence of baseline erosions to be more strongly linked to future erosive damage compared with the presence of anti-CCP [30]. In contrast Karlson et al. in the Brigham Rheumatoid Arthritis Sequential Study (BRASS) showed by multivariable analysis that SE status was strongly associated with the presence of anti-CCP (OR 1.81, 95% CI 1.24–2.66) [32]. Although SE was independently associated with an erosive phenotype, this was not significant after conditioning for anti-CCP, suggesting that the presence of anti-CCP may represent a “causal pathway” for predicting erosions.

The presence of baseline erosions was shown to be a predictor of future joint damage in five studies included in this review [19, 25, 30, 36, 38]. One of these (a cohort study of 112 patients) examined the SvdH score over a period of 10 years by univariate analysis. The presence of ACPA was found to be significantly correlated to radiographic score at baseline [19]. Further analysis identified baseline erosion score to be the most important independent prognostic factor of the total erosion score at ten years, ACPA positivity being the next important.

Finally these studies also reconfirm the superiority of ACPA over RF as independent predictors of joint damage in RA [30–32].

## 4. Discussion

Most systematic reviews carried out to date have concentrated on the diagnostic value of ACPA. There has been some reference to prognosis in these studies, but the majority of these have focused on anti-CCP. This review examines studies looking at all the currently available ACPA specificities and confirms their prognostic value as predictors of joint damage in RA.

The results of individual studies published so far have been conflicting. This could be as a result of the inherent heterogeneity of these studies and in particular as a result of different study designs and length of follow-up. However our meta-analysis clearly shows the value of ACPA, in particular anti-CCP, and anti-MCV in predicting the severity of disease as measured by joint damage.

Other ACPA that have also been studied are anti-CFFCP1 and anti-CEP1, both of which were shown to be predictive of joint erosions. The ACPA predominantly analysed for this outcome were anti-CCP. The next most frequently studied ACPA was anti-MCV. There was only one study of relevance on anti-CFFCP1 and a recent concise report on the predictive value of anti-CEP1. The presence of ACPA and their concentration at baseline was strongly predictive of radiographic progression in all but one study. This was most apparent in studies that had a long follow-up duration, showing a positive correlation with length of follow-up. ACPA predict disease prognosis and can be useful in devising appropriate treatment strategies for patients who are at risk of developing RA. This would optimize disease management, by reducing morbidity, and would also help utilize health resources in a cost-effective manner.

This review highlights the superiority of anti-MCV over anti-CCP in predicting joint erosions. The evidence on other ACPA lacks robust long-term follow-up studies. We note that the means of detection of erosions has largely been limited to the use of conventional radiography with only two studies using MRI. High resolution sonography and MRI have been proven to be superior to plain X-rays and need to be used as the standard means to detect joint damage [41]. Perhaps more widespread use of these modalities in detecting joint damage should be part of our clinical practice.

It has been shown that the presence of the shared epitope and the extent of epitope spreading both contribute to the strength of ACPA in predicting joint erosions [2, 23]. Recent work has suggested that anti-CarP (antibodies to carbamylated proteins) [42] may have a pathogenic role in RA, with a predictive role in disease outcome. However they are found more frequently in ACPA positive rather than ACPA negative RA. Whether or not these novel autoantibodies are independent predictors of joint damage in RA is yet to be proven.

We already know from previous studies that ACPA are an important indicator in the diagnosis of RA. Our study concludes that ACPA have a valuable role in determining the prognosis of RA. They are strongly predictive of the development of erosions. There are other predictors, but ACPA represent a particularly useful investigation in the routine screening of patients with an inflammatory arthritis. The presence of these antibodies identifies a more aggressive form of disease and helps to justify early treatment escalation.

## Figures and Tables

**Table 1:** Studies included in the systematic review.

Author	Year	Study type	Cohort size	Study duration in years	Anti-CCP	Other ACPA	Erosions	SvdH	Larsen
Fisher et al. [23]	2011	Prospective	408	5	50%	27% (anti-CEP1)			8
Mansour et al. [24]	2010	Prospective	123	2		42% (anti-MCV)	77%		
Hetland et al. [25]	2010	Double-blind randomized	110	5	58%			13	
Plant and Thomson [26]	2010	Prospective	269	5	56%		48%		10
Kim et al. [27]	2010	Longitudinal	216	2	82%		75%		
Sanmartí et al. [28]	2009	Randomized controlled	322	2	74%	83% (anti-CFFCP)	70%		7
Rojas-Villarraga et al. [20]	2009	Prospective	157	3	79%			6	
van der Linden et al. [29]	2009	Longitudinal	687	5	67%	92% (anti-MCV)		48	
Syversen et al. [21]	2010	Longitudinal	125	10	63%	64% (anti-MCV)	54%	50	
Reneses et al. [30]	2009	Prospective	134	1	54%		36%		
Innala et al. [10]	2008	Randomized controlled	210	2	86%	96% (anti-MCV)	66%		12
Courvoisier et al. [31]	2008	Prospective	112	10	58%		70%	46	
Mathsson et al. [22]	2008	Randomized controlled	273	2	58%	71% (anti-MCV)	71.00%		18
Bukhari et al. [18]	2007	Controlled cross-sectional	427	5	70%		79%		29
Karlson et al. [32]	2008	Prospective	689	5	67%		59%		
Machold et al. [17]	2007	Controlled cross-sectional	55	3	63%		65%		75
Mewar et al. [33]	2006	Prospective	872	3	77%		77%		49
Kaltenhäuser et al. [19]	2007	Prospective	126	6	69%		65%		29
Meyer and Nicaise-Roland [34]	2006	Prospective	172	3	64%		57%	5	
Boire et al. [35]	2005	Prospective	165	2	53%	48% (anti-MCV)	64.00%	34	
Bongi et al. [36]	2004	Controlled cross-sectional	89	1	88%		49%		
Lindqvist et al. [37]	2005	Prospective	186	10	80%		83%		37
Jansen et al. [38]	2003	Prospective	289	2	65%		34%	19	
Orbach et al. [39]	2002	Prospective	101	1	59%		63%		
Vencovský et al. [40]	2003	Prospective	104	2	47%		64%		18

Erosions: the percentage of patients that showed progressive erosive change.

SvdH: the highest Sharp score recorded, with asterisk the change in Sharp score.

Larsen: the highest Larsen score recorded.

## Abbreviations

ACPA: Anticitrullinated protein antigens
CCP: Cyclic citrullinated peptide
CEP1: Citrullinated enolase peptide 1
CFFCP1: Chimeric fibrin/filaggrin citrullinated peptide 1
MCV: Modified citrullinated vimentin
PAD: Peptidyl arginine deiminase
SE: Shared epitope
SvdH: Sharp van der Heijde
Anti-CarP: Anticarbamylated protein
SEN: Simple erosion narrowing
PTNP22: Protein tyrosine phosphate type 22.
